# Influence of Galloyl Moiety in Interaction of Epicatechin with Bovine Serum Albumin: A Spectroscopic and Thermodynamic Characterization

**DOI:** 10.1371/journal.pone.0043321

**Published:** 2012-08-20

**Authors:** Sandip Pal, Chabita Saha, Maidul Hossain, Subrata Kumar Dey, Gopinatha Suresh Kumar

**Affiliations:** 1 School of Biotechnology and Biological Sciences, West Bengal University of Technology, Kolkata, India; 2 Biophysical Chemistry Laboratory, Chemistry Division, CSIR – Indian Institute of Chemical Biology, Kolkata, India; Russian Academy of Sciences, Institute for Biological Instrumentation, Russian Federation

## Abstract

The health benefits stemming from green tea are well known, but the exact mechanism of its biological activity is not elucidated. Epicatechin (EC) and epicatechin gallate (ECG) are two dietary catechins ubiquitously present in green tea. Serum albumins functionally carry these catechins through the circulatory system and eliminate reactive oxygen species (ROS) induced injury. In the present study ECG is observed to have higher antioxidant activity; which is attributed to the presence of galloyl moiety. The binding affinity of these catechins to bovine serum albumin (BSA) will govern the efficacy of their biological activity. EC and ECG bind with BSA with binding constants 1.0×10^6^ M^−1^ and 6.6×10^7^ M^−1^, respectively. Changes in secondary structure of BSA on interaction with EC and ECG have been identified by circular dichroism (CD) and Fourier transform infrared (FT-IR) spectroscopy. Thermodynamic characterization reveals the binding process to be exothermic, spontaneous and entropy driven. Mixed binding forces (hydrophobic, electrostatic and hydrogen bonding) exist between ECG and BSA. Binding site for EC is primarily site-II in sub-domain IIIA of BSA and for ECG; it is site-I in sub-domain IIA. ECG with its high antioxidant activity accompanied by high affinity for BSA could be a model in drug designing.

## Introduction

**Figure 1:**
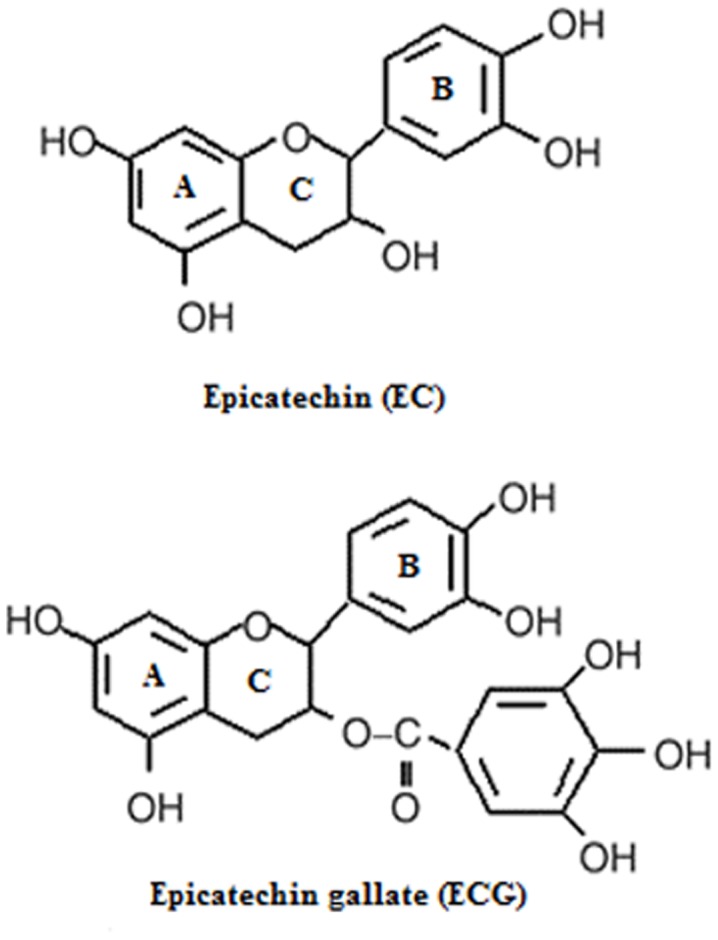
The chemical structure of epicatechin (EC) and epicatechin gallate (ECG).

Diets rich in antioxidants contribute to lower incidence of several major chronic diseases. In particular, cancer development or growth is inhibited by antioxidants. Antioxidants delay or prevent the oxidation of a given substrate by free radicals. Epicatechin (EC) and epicatechin gallate (ECG) are ubiquitous antioxidants present in green tea. These catechins have been investigated for various applications stemming from their antioxidant properties, like radioprotective properties [1] and antioxidative effect [2]. These two catechins are considered under the flavan-3-ol group of flavonoids. Other flavonoids present in green tea, are epigallocatechin (EGC) and epigallocatechin gallate (EGCG) [3]. ECG is the gallic acid ester form of EC as represented in Fig. 1. It is evident from the structure that the main difference between EC and ECG arises from the absence or presence of galloyl moiety at the position three of the C-ring. The chemistry of the C-ring as well as the number and distribution of hydroxyl groups and their substitutions determine the antioxidant activity of these flavonoids in general [4], [5].

Interactions of flavonoids and proteins have gained lot of attention recently [6]–[9] as serum albumins are the major soluble protein constituent of the circulatory system. Dietary flavonoids on consumption first interact with albumin protein of blood for transport to different tissues of our body. Bovine serum albumin (BSA) is one of the most extensively studied protein, particularly because of its structure homology with human serum albumin (HSA) [10]. BSA interacts with wide range of chemicals such as sanguinarine, quercetin, rutin, gemcitabine hydrochloride and proflavin [11]–[14]. Interaction of BSA with dietary flavonoids has revealed that the binding efficiency is governed by the structural arrangement and the resonance between the rings A and B is reported to be essential for the antioxidant and biological activity of these flavonoids [15]. The measurement of the antioxidant activity of EC and ECG would establish the contribution of galloyl moiety to the antioxidant activity. The interaction of these two molecules with BSA would shed light on the molecular basis of rational designing of new and more efficient therapeutic agents that can recognize and bind to specific biological targets for improved drug activity.

In the present study interactions of EC and ECG with BSA were explored by fluorescence, circular dichroism (CD) and Fourier transform infrared (FT-IR) spectroscopy. Binding affinities and the accompanied structural changes during the binding process were measured. The binding site was identified using competition method with warfarin and ibuprofen, whose binding site to BSA is known. Binding mode and thermodynamic characterization of the binding process was done by isothermal microcalorimetry (ITC).

## Materials and Methods

### Biochemicals

BSA, EC, ECG, warfarin, ibuprofen and DPPH (2, 2-diphenyl-1-picryl hydrazyl) were obtained from Sigma-Aldrich, St. Louis, MO, USA. All the experiments were carried out in 50 mM phosphate buffer of pH 6.7.

**Figure 2:**
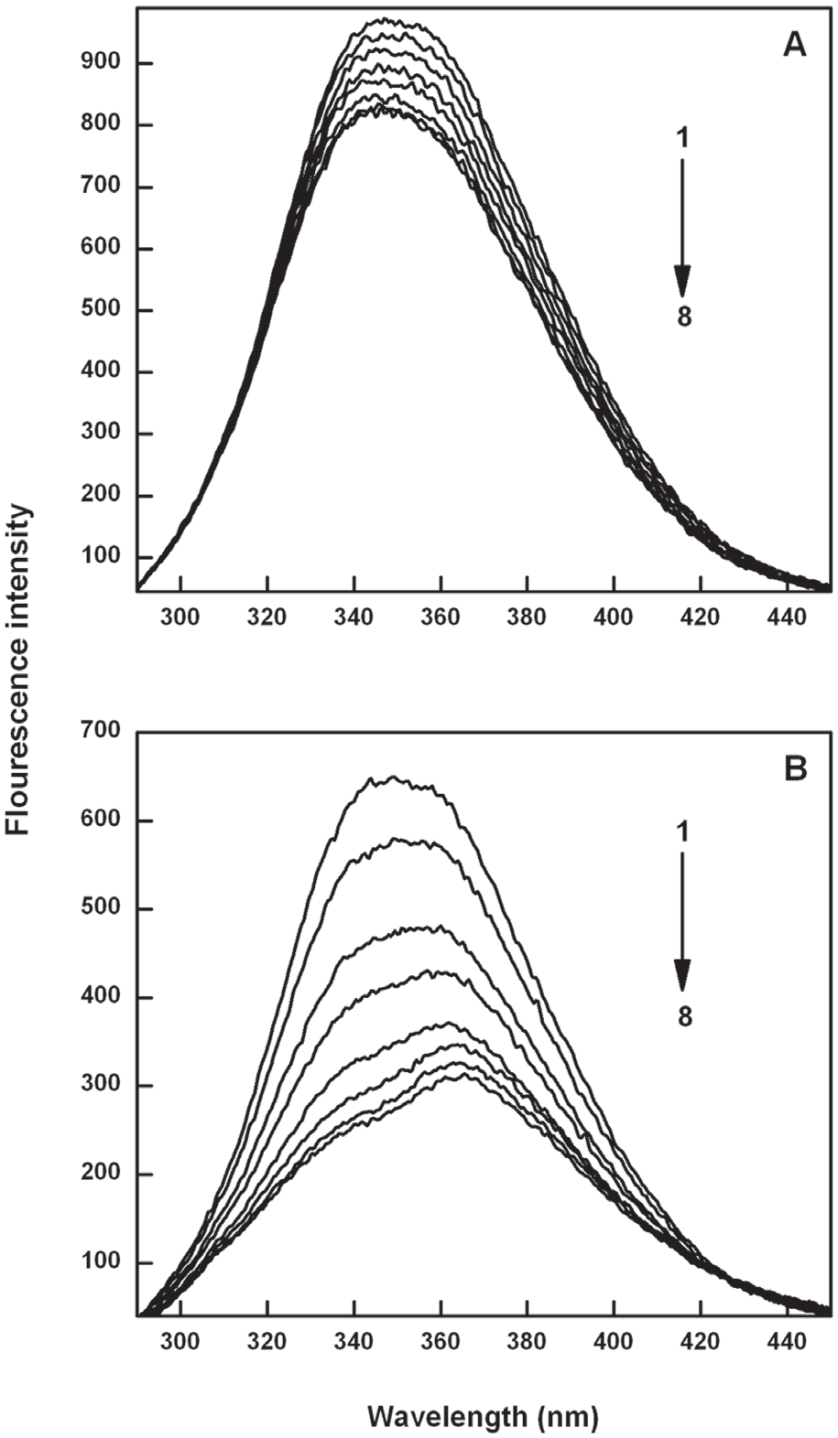
The fluorescence emission spectra of Catechin-BSA system (T = 298 K, λ_ex_ = 280 nm). Panel (A) curves (1–8) denote 0 to 0.375 µM of EC and panel (B) curves (1–8) denote 0 to 0.5 µM of ECG.

### Antioxidant Activity

Scavenging ability of EC and ECG on DPPH radical was measured according to the method reported by Turkmen et al. [16] with a slight modification. Solutions containing EC and ECG (1 µM−1000 µM) were allowed to scavenge 0.6 mM DPPH and the absorbance of remaining DPPH was measured at 517 nm using methanol as a blank. The spectrophotometric analyses were performed at room temperature using matched quartz cells of 1 cm path length with the help of Varian UV-Visible Spectrophotometer (CARY 100 Bio, USA). Antioxidant activity (AA) was expressed as percentage inhibition of the DPPH radical and was determined by the following equation:

AA (%) = (control absorbance – sample absorbance)/control absorbance × 100

The inhibitory concentration 50 (IC_50_) values were calculated from data obtained graphically, using mathematical method based on the principle of the right-angled triangle: IE_50_ = D−[(A−50% maximum response)X]/Y, in which A is the immediately higher response of 50% maximum response; B is the immediately lower response of 50% maximum response; D = log concentration corresponding to A response; C = log concentration corresponding to B response; X = D−C; and Y = A−B [17].

**Table 1:** Binding constants of catechins (EC and ECG) to BSA in presence of site markers warfarin and ibuprofen at 25°C.

Catechins	Site Marker	Binding constant (×10^6^ M^−1^)
EC	Blank	1.0
	Warfarin	0.55
	Ibuprofen	0.27
ECG	Blank	66.0
	Warfarin	8.9
	Ibuprofen	23.0

### Fluorescence Spectroscopy

All the fluorescence measurements were made on a Perkin Elmer fluorescence spectrophotometer (LS-55) equipped with 150 W Xenon flash lamp and using fluorescence-free quartz cell of 1 cm path length at room temperature [18]. In all experiments the fluorescence of BSA (2.5×10^−6^ M) in 50 mM phosphate buffer of pH 6.7 was followed on excitation at 280 nm.

**Figure 3:**
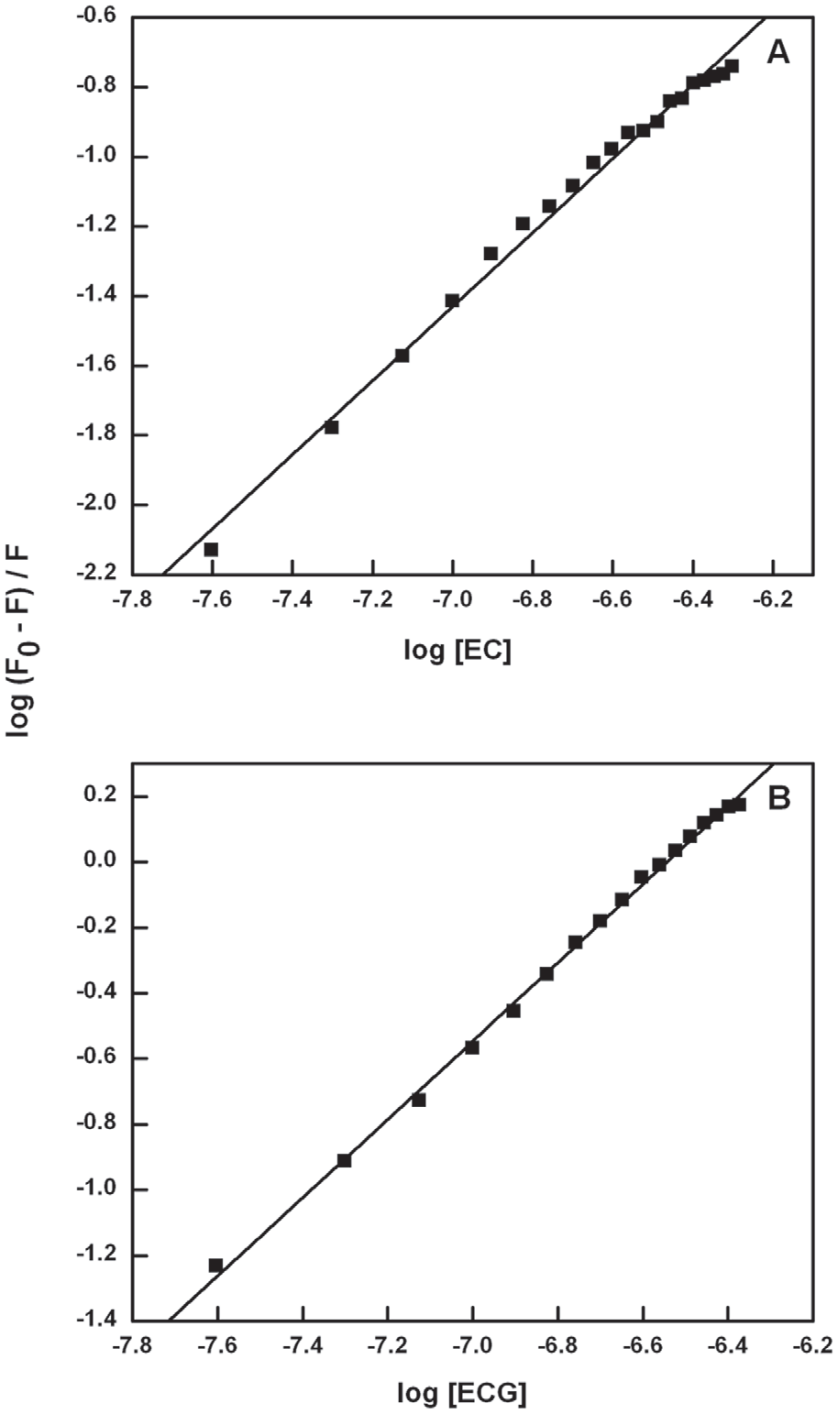
Double logarithmic plot of Catechin-BSA system (T = 298 K, λ_ex_ = 280 nm). Panel (A) shows EC quenching BSA fluorescence and panel (B) shows ECG quenching BSA fluorescence.

### Fluorescence Quenching Studies

Solution containing 2.5×10^−6^ M BSA in 50 mM phosphate buffer of pH 6.7 was titrated with successive addition of catechins EC (1×10^−5^ M) and ECG (1×10^−5^ M) separately. The excitation wavelength was 280 nm and the emission spectra were recorded from 290 nm to 450 nm. The intensities of emission maxima were used for the calculation of binding constants using Eq. 1 (vide infra).

### Warfarin and Ibuprofen Displacement Studies

To identify the binding sites of EC and ECG in BSA, displacement of warfarin and ibuprofen known to bind at site I and site II, respectively, was followed fluorimetrically. Equimolar solution of BSA and warfarin (2.5×10^−6^ M each) was mixed and kept for 1 hr and titrated with EC (1×10^−5^ M) and ECG (1×10^−5^ M) in separate experiments. Similar solution containing equimolar ibuprofen and BSA was prepared and titrated with EC and ECG separately to identify the binding sites of EC and EG with BSA. Titration was followed fluorimetrically using excitation wavelength of 280 nm.

### Circular Dichroism (CD) Spectroscopy

All the CD experiments were carried out on a Jasco-815 automatic spectropolarimeter (Jasco International Co., Ltd. Hachioji, Japan) equipped with a peltier cuvette holder and temperature controller PFD425 L/15. The BSA concentration and path length of the cuvette used were 1 µM and 0.1 cm, respectively. The instrument parameters were set at scanning speed of 50 nm/min, bandwidth of 1.0 nm and sensitivity of 100 milli degree. CD spectra of BSA in presence of EC and ECG in various D/P ratios were recorded separately. Four scans were averaged and smoothed to improve signal to noise ratio. The molar ellipticity values are expressed in terms of mean residue molar ellipticity, in units of deg. cm^2^ dmol^−1^ [11], [18]. Secondary structure analysis was performed by the software supplied by Jasco.

**Figure 4:**
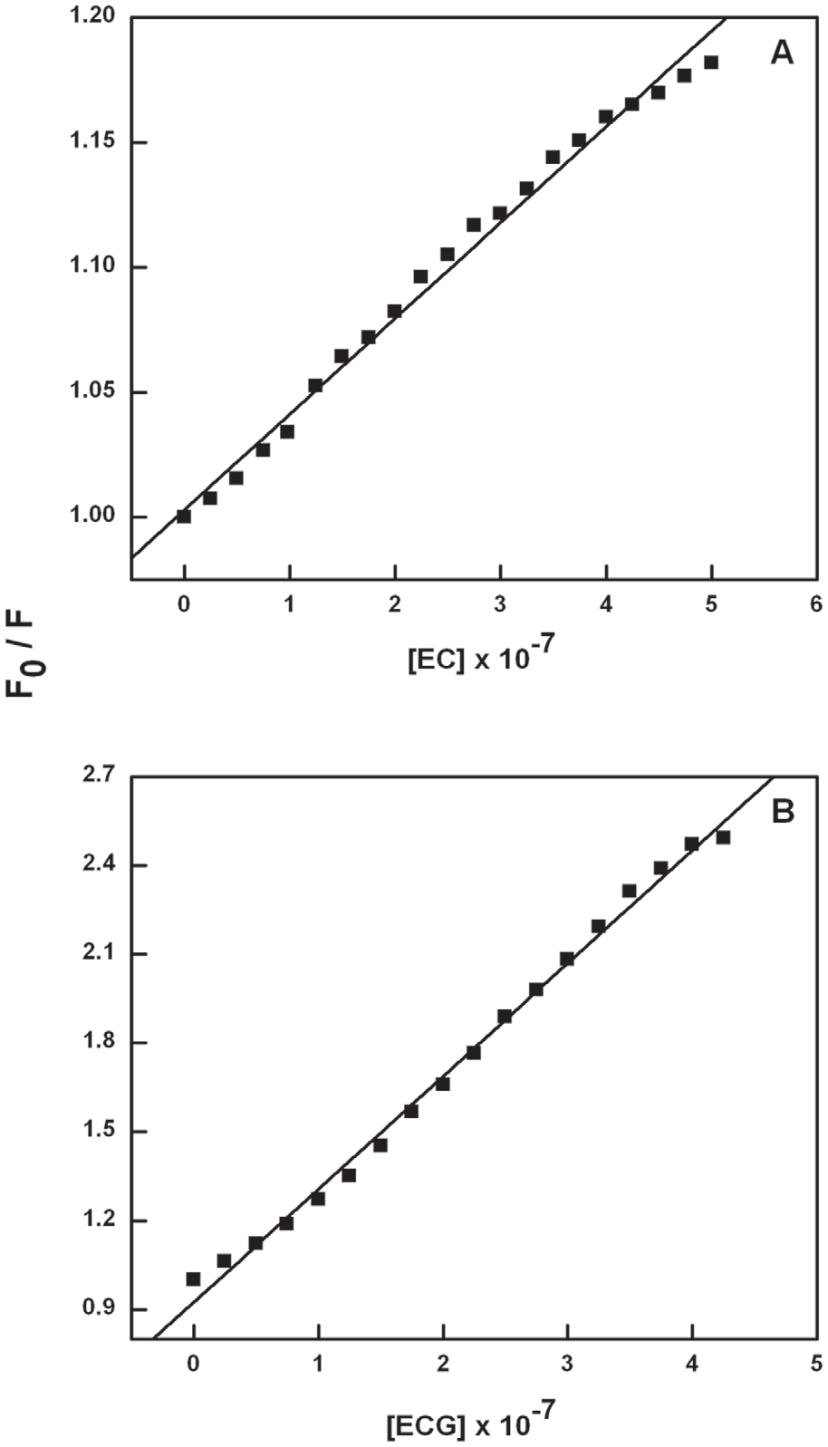
Stern-Volmer plot for Catechin-BSA system (T = 298 K). Stern-Volmer plot for the binding of EC with BSA (A) and ECG with BSA (B).

**Table 2:** Stern-Volmer (K_sv_) quenching constants for BSA and ECG interaction under various conditions, at 25°C.

Samples	K_sv_ (×10^6^ M^−1^)
BSA	3.8
BSA + NaCl (0.1 M)	0.37
BSA + NaCl (0.3 M)	0.35
BSA + NaCl (0.5 M)	0.22
BSA + Sucrose (1.0 M)	0.19

### FT-IR Spectroscopic Measurements

FT-IR measurements were carried out on Perkin Elmer, Spectrum GX equipment using ZnSe window [19], [20]. Hundred scans were recorded for each sample in the spectral range of 400–4000 cm^−1^ with a resolution of 4 cm^−1^. The background was corrected before scanning the samples and the buffer spectrum collected. FT-IR spectra of free BSA (1×10^−4^ M), BSA-EC complex (1∶1 and 1∶2) and BSA-ECG complex (1∶1 and 1∶2) were recorded to identify changes in secondary structure in BSA on interaction with EC and ECG. All the experiments were performed at room temperature (25°C).

### Isothermal Titration Calorimetry

The energetics of the binding of catechins to BSA was studied by isothermal titration calorimetry (ITC) using a VP ITC unit (MicroCal, Northampton, MA, USA) with calorimeter cell volume of 1.423 ml. All solutions were degassed under vacuum (140 mbar, 10 min) on the MicroCal’s Thermovac unit to eliminate air bubble formation inside the calorimeter cell. Briefly, the calorimeter syringe was filled with a concentrated solution of EC or ECG (500 µM each). Successive injections of 10 µl of this solution into 50 µM solution of BSA, in the same buffer contained in the calorimeter cell was effected from the rotating syringe that enabled constant stirring of the solution. The data were corrected for the heat of dilution of catechins, which was determined in a separate set of experiments under identical conditions. The titration and analysis were performed through Origin 7 software provided with the unit.

**Figure 5:**
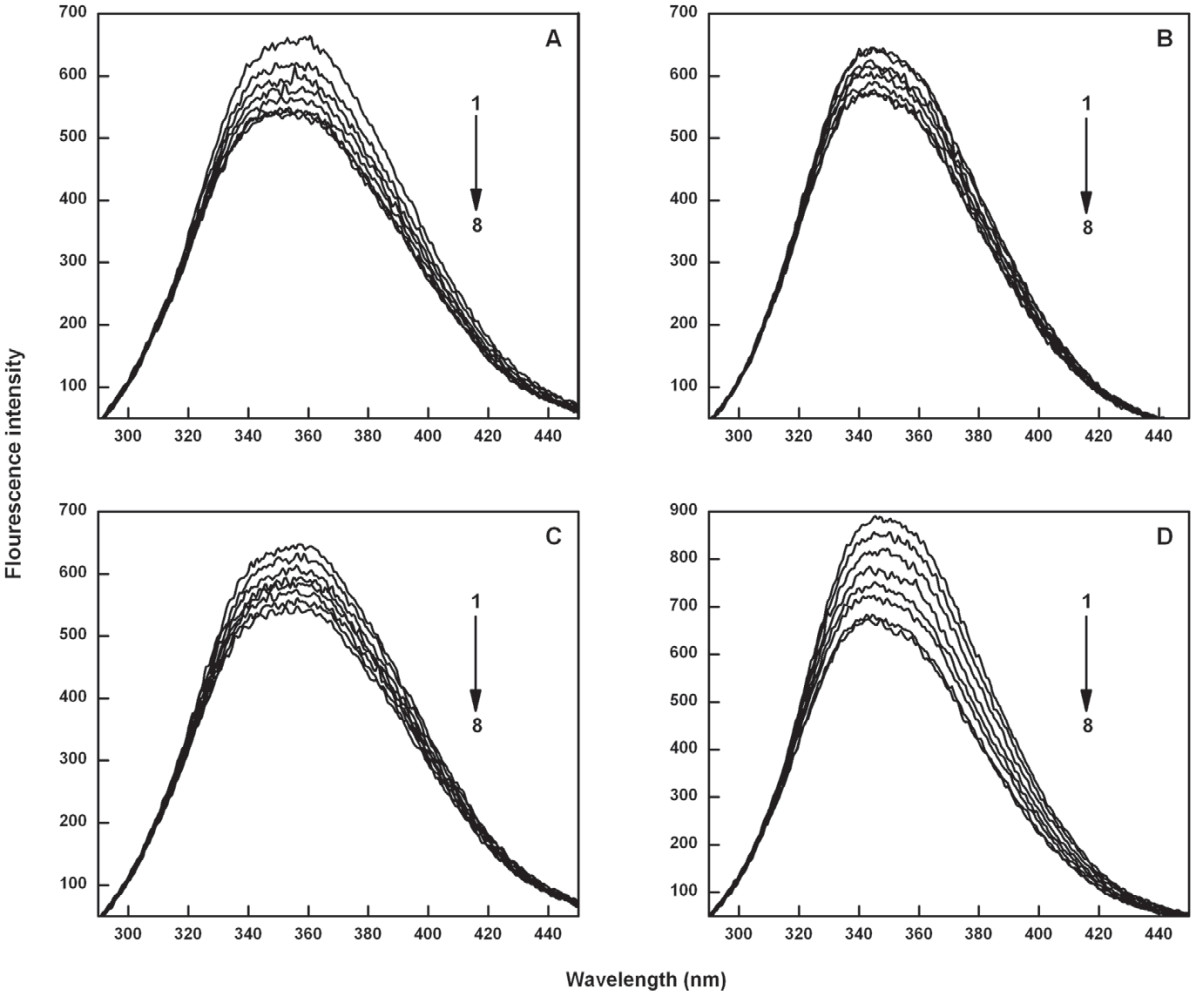
Effect of site marker on the Catechin-BSA complex (T = 298 K, λ_ex_ = 280 nm). Effect of warfarin (A) and ibuprofen (B) on quenching of BSA with EC. Effect of warfarin (C) and ibuprofen (D) on quenching of BSA with ECG.

**Figure 6:**
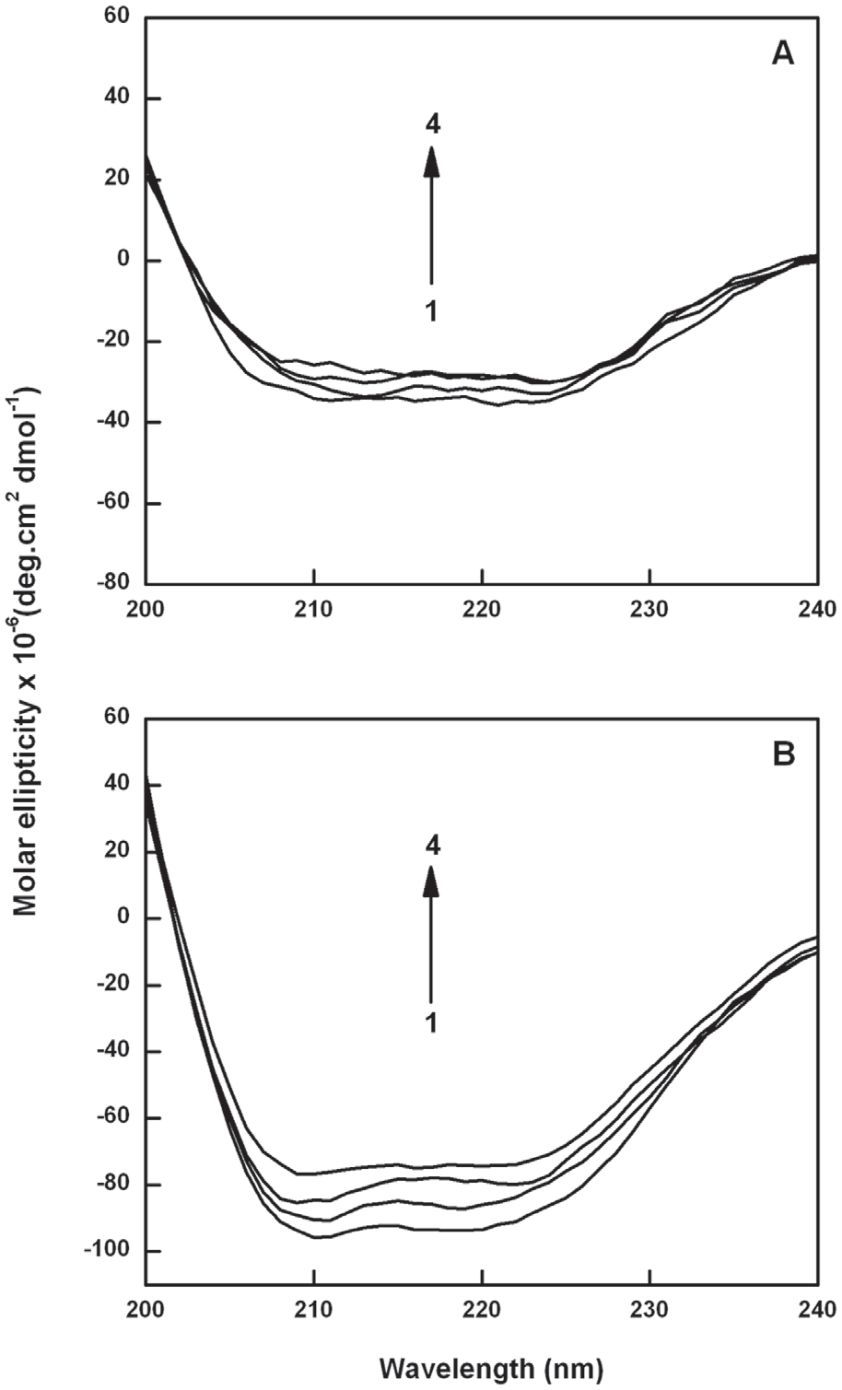
The CD spectra of Catechin-BSA system (T = 298 K). Panel (A) curves (1–4) denote 0, 1, 4 and 8 µM of EC and panel (B) curves (1–4) denote 0, 1, 2 and 3 µM of ECG.

**Table 3:** Variation of the secondary structure of BSA in presence of different concentrations of catechins (EC and ECG) at 25°C.

Catechins	D/P	Alpha helix (%)	Beta sheet (%)
EC	0	58.5	30.1
	1	58.1	30.5
	4	54.7	32.5
	8	53.5	33.1
ECG	0	58.8	30.5
	1	51.7	34.1
	2	50.9	35.6
	3	49.9	38.9

## Results and Discussion

### Antioxidant Activity

The IC_50_ values for the antioxidant activity of EC and ECG are 475 µM and 120 µM, respectively. From these values it is evident that ECG is more efficient in scavenging the DPPH free radicals than EC, and thus a better antioxidant. The antioxidant activity of ECG is attributed to the galloyl group containing three phenolic groups capable of transferring hydrogen easily to the oxidizing agent. Higher antioxidant activity combined with higher binding efficacy (discussed later) with BSA would be effective in efficient transportation and combating reactive oxygen species (ROS) induced stress.

### Characteristics of the Fluorescence Spectra

There are two tryptophans (Trp 134 and Trp 213) in BSA of which Trp 134 is located on the surface and Trp 213 resides in the hydrophobic pocket of the protein molecule [21]. These two tryptophan residues account for the major fluorescence emission intensity of BSA [22]. The interaction of EC and ECG with BSA was evaluated by measuring the intrinsic fluorescence intensity changes of BSA upon the addition of EC and ECG in independent experiments. The fluorescence emission spectrum of BSA as shown in Fig. 2 has an emission maximum at 350 nm when excited at 280 nm. On progressive addition of various concentrations of EC and ECG to BSA, the emission intensity decreases as shown in Fig. 2A and Fig. 2B, respectively. Quenching of the fluorescence intensity of BSA imply that the catechins bind at close proximity to the tryptophan and tyrosine residues and molecular arrangement of BSA is affected by the interactions. Progressive red shift of fluorescence emission of BSA was observed with increasing concentration of ECG, while no such peak shift was observed for BSA-EC interaction. The spectral shift observed for ECG is attributed to its complexation with BSA; resulting in change in the microenvironment of the tryptophan and tyrosine residues of the BSA [6] and the secondary structure of BSA. Absence of such spectral shifts in EC emphasize that galloyl group strongly influence the binding affinity of catechins to BSA. The fluorescence of EC and ECG on excitation at 280 nm did not contribute to the fluorescence intensity of BSA (data not shown).

### Analysis of Binding Parameters

Albumins have high affinity for fatty acids, hematin, bilirubin and a broad affinity for small negatively charged aromatic compounds. Serum albumins facilitate the disposition and transport of variety of ligands like quercetin and other flavonoids [23], [24]. Recent studies have suggested that the catechins form complexes with BSA for transportation in blood, and their binding affinity for albumin is believed to modulate their bioavailability [25]. Fluorescence emission spectra of BSA (2.5×10^−6^ M) as shown in Fig. 2B on titrating with ECG (1×10^−5^ M) shows progressive red shift in λ_max_, evidencing formation of complex between BSA and ECG, leading to change in micro environment of the fluorophore. Such peak shift was not observed for BSA-EC interaction suggesting weaker interactions (Fig. 2A). Binding affinity of EC and ECG with BSA in terms of binding constants was determined using the following equation [26]:
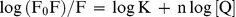
(1) where K is the binding constant and n is the number of binding sites, F_0_ is the fluorescence intensity of free BSA and F is the consecutive fluorescence on addition of catechins. Plot of log (F_0_– F)/F against log [Q] was used to determine the values of K and n from the intercept and the slope respectively. The binding constants (K) for BSA-EC and BSA-ECG complexes are 1.0×10^6 ^M^−1^ and 6.6×10^7^ M^−1^, respectively, and are summarized in Table 1 and respective plots are showed in Fig. 3A and 3B. The values of n are found to be approximately equal to 1 (one) for both the catechin-BSA complexes indicating that there is only one binding site involved in each of the cases. In the light of these findings it can be reasoned that the binding affinity to BSA is higher for galloylated catechin than the non-galloylated one; emphasizing the biological functional activity of the galloyl moiety attached to the C-ring of the flavonoid. The three phenolic and one carboxyl group in the galloyl moiety can enhance the hydrogen bonding between BSA and ECG.

**Figure 7:**
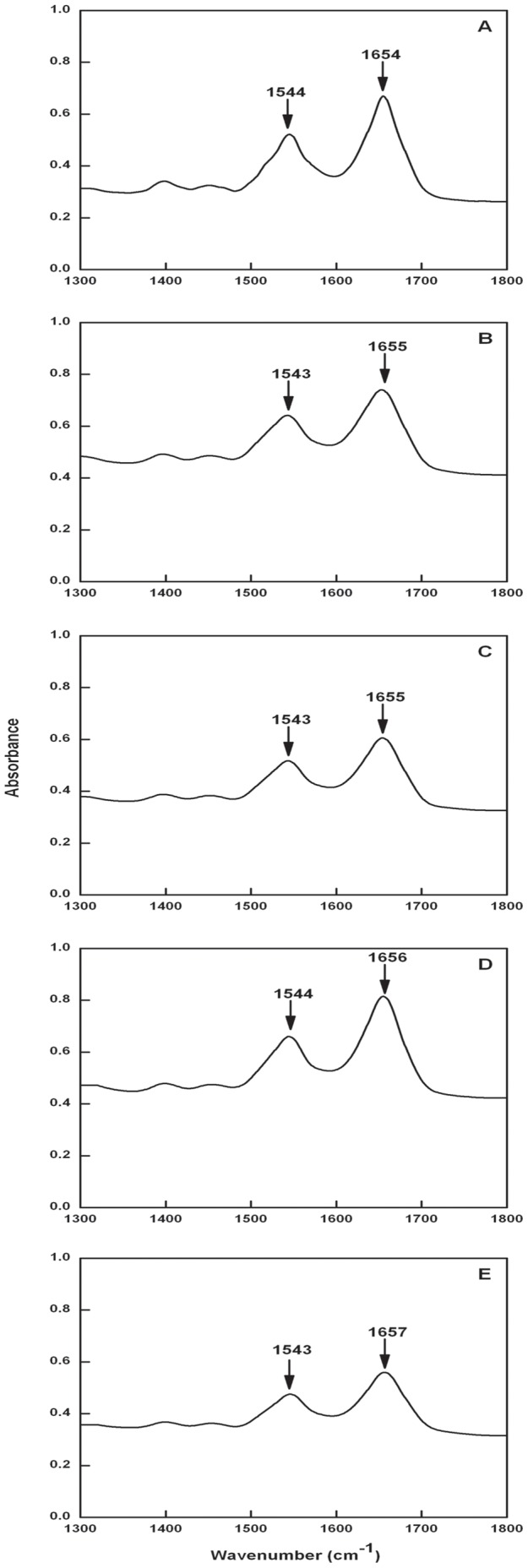
The FT-IR spectra of BSA-Catechin system (T = 298 K). Panel (A) curve denote free BSA and panel (B), (C), (D) and (E) curves denote BSA-EC (1∶1), BSA-EC (1∶2), BSA-ECG (1∶1) and BSA-ECG (1∶2) respectively in the region of 1300–1800 cm^−1^. The values in brackets denote molar ratios of the respective BSA-catechin complexes.

### Analysis of Quenching Parameters

The fluorescence emission intensity of BSA (2.5×10^−6^ M) was quenched upon addition of EC (1×10^−5^ M) and ECG (1×10^−5^ M) in separate experiments. The fluorescence emission quenching data were analyzed by the Stern-Volmer equation:
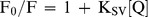
(2) where F_0_ and F are the steady-state fluorescence intensities in the absence and presence of quencher, K_SV_ is the Stern-Volmer quenching constant and [Q] is the concentration of quencher (here EC or ECG). The Stern-Volmer quenching constant (K_SV_) values for EC and ECG are 3.7×10^5^ M^−1^ (R = 0.9951) and 3.8×10^6^ M^−1^ (R = 0.9973), respectively, (Fig. 4A and 4B) and tabulated in Table 2. Higher K_SV_ for BSA-ECG is good measure of the strong ground state binding compared to EC. The quenching rate constants (K_q_) were evaluated using the equation:
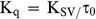
(3) where τ0 is the average lifetime of the protein without the quencher. The average lifetime of the biopolymer is 10−8 s [27]. Hence, the quenching rate constants (Kq) for the interaction of EC-BSA and ECG-BSA were calculated to be 3.7×1013 M^−1^ s^−1^ and 3.8×1014 M^−1^ s^−1^, respectively. The higher KSV and Kq values for the ECG-BSA system signify that ECG quenches BSA through complexation more efficiently than EC.

### Effect of Ionic Strength on the Binding of ECG to BSA

The salt dependence of a biomolecular association is often used to access the contribution of charge-charge interaction to the free energy of binding. To understand the role of electrostatic interactions in the binding process, the ionic strength dependence of the binding of EC and ECG with BSA was studied. Quenching of BSA (2.5×10^−6^ M) fluorescence emission intensity by ECG (1×10^−5^ M) was measured in solutions of varying ionic strength (0.1 M –0.5 M) using NaCl and K_SV_ values were calculated. The K_SV_ value is 3.8×10^6^ M^−1^ in absence of NaCl and this value decreased with increase in ionic strength to 3.7×10^5^, 3.5×10^5^ and 2.2×10^5^ M^−1^ for 0.1, 0.3 and 0.5 M NaCl, respectively (Table 2), implicating electrostatic attraction as one of the binding forces of ECG with BSA.

**Figure 8:**
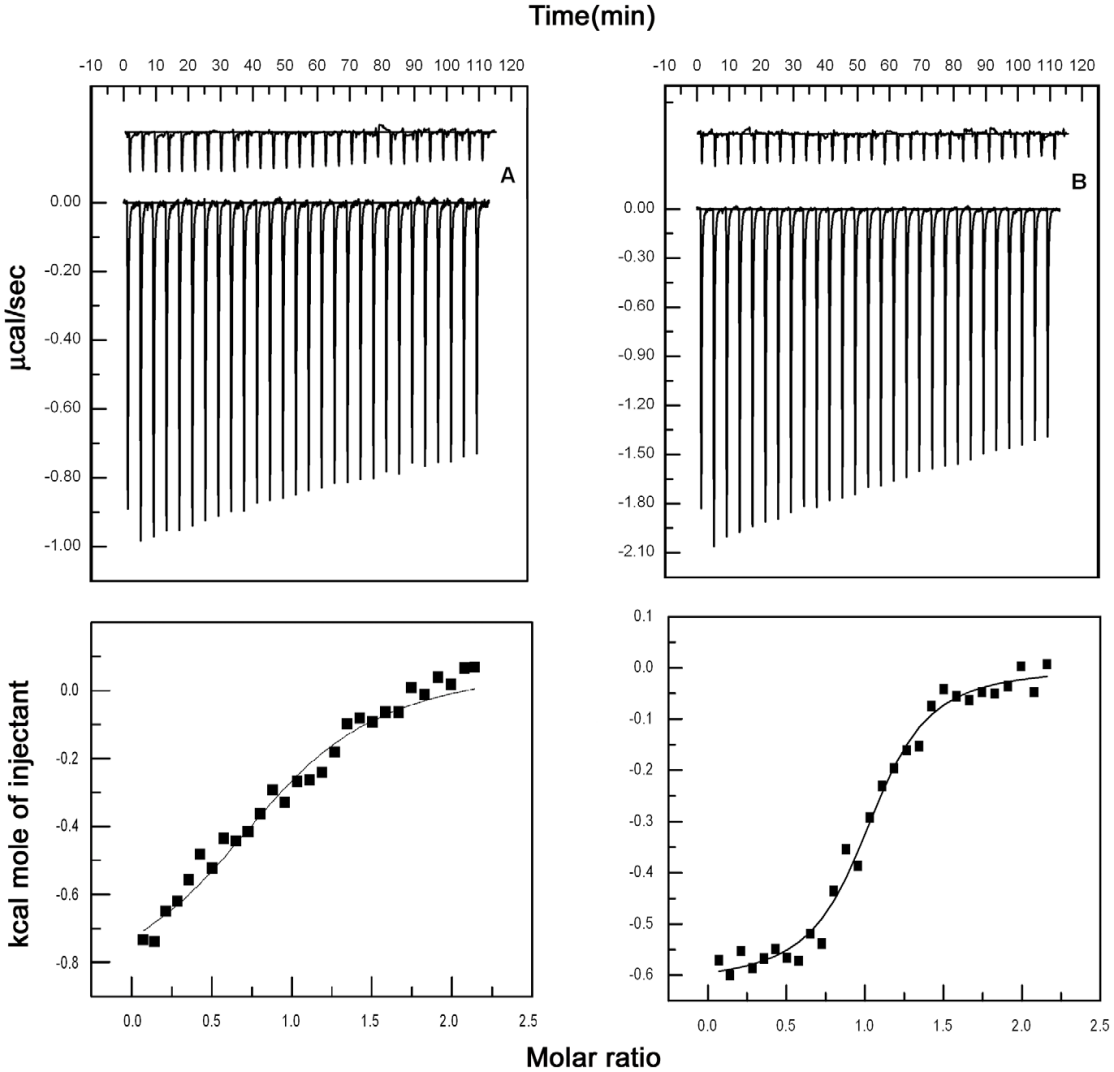
ITC profiles of BSA when titrated with catechins (T = 298 K). The top panels (A) and (B) present raw results for the sequential injection of EC (500 µM) and ECG (500 µM) into BSA solution (50 µM) and dilution of catechins into buffer (curves on the top offset for clarity). The lower panels show the integrated heat results after correction of heat of dilution against the mole ratio of Catechin/BSA. The points (closed squares) were fitted to a one-site model and the solid lines represent the best-fit results.

**Table 4:** Thermodynamic parameters for catechin-BSA system at 25°C.

Catechins	Number of binding sites	Binding constant (×10^4^ M^−1^)	Δ*G*° (cal/mol)	Δ*H*° (cal/mol)	*T*Δ*S*° (cal/mol)
EC	0.937	9.68±0.19	−6809.5±65	−968.7±65	5840.8
ECG	1.03	27.0±0.46	−7409.1±13	−614.7±13	6794.4

### Effect of Sucrose on the Binding of ECG to BSA

Studies on catechin-BSA interactions in the presence of sucrose will provide information on the involvement hydrogen bonding process since sucrose has hydroxyl groups which can interfere in the hydrogen bond formation between the drug and protein. Quenching of BSA (2.5×10^−6^ M) fluorescence emission intensity by ECG (1×10^−5^ M) was followed in presence of 1 M sucrose; known for formation of hydrogen bonds through its hydroxyl groups [28]. The Stern-Volmer quenching constant (K_SV_) was measured to be 1.9×10^5^ M^−1^ which is more than an order less than that observed in absence of sucrose (Table 2). From the findings it is inferred that hydrogen bonding contributes to stabilization of BSA-ECG complex.

### Site Selective Binding of EC and ECG

The capability of serum albumins to bind to aromatic and heterocyclic compounds is largely dependent on the existence of two major binding regions, namely site-I and site-II [29]–[31] located within specialized cavities in sub-domains IIA and IIIA, respectively [32], [33]. Both these domains are characterized by the presence of a central cavity formed from six amphiphatic helices arranged in a myoglobin like fold. Warfarin and ibuprofen are fluorescence probes whose primary binding site to BSA are known and are referred to as site-I and site-II, respectively. Competitive binding experiments reveal that EC has higher specificity for site-II where ibuprofen binds primarily and ECG binds preferentially to site-I where it competes with warfarin. The emission spectra of BSA (2.5×10^−6^ M) in presence of warfarin and ibuprofen (2.5×10^−6^ M) are shown in Fig. 5A, 5C and 5B, 5D, respectively. The spectral profiles are different from free BSA as shown in Fig. 2. When complexed with warfarin a slight red shift in the λ_max_ to 363 nm, is observed whereas a slight blue shift to 342 nm is observed for BSA-ibuprofen complex. On addition of ECG to the solution containing BSA and warfarin, no further change in spectral profile (Fig. 5C) was observed suggesting that ECG is unable to bind to site-I; which is its preferred binding site. This is confirmed when significant quenching was observed of BSA-ibuprofen complex by ECG (Fig. 5D) where site-I is free. On the other hand, on addition of EC (Fig. 5A) small amount of quenching was observed in BSA-warfarin emission profile, suggesting that the preferred binding is at site-II. This is ascertained when negligible quenching was observed in emission intensity of BSA-ibuprofen complex on addition of EC (Fig. 5B). Here the chemical and structural difference of EC and ECG is reflected in their preference for site-I and site-II of BSA respectively. The binding constants of EC and ECG to BSA in presence of warfarin and ibuprofen are summarized in Table 1.

### Circular Dichroism Spectroscopy Study

Circular dichroism (CD) is a sensitive technique to monitor the conformational changes leading to protein unfolding upon interaction with the ligand. The CD spectra for BSA-EC (0–8 µM of EC) and BSA-ECG (0–3 µM of ECG) system are represented in Fig. 6A and 6B, respectively. The far ultraviolet CD spectrum of BSA showed two negative minima at 209 and 222 nm which is characteristic of α-helical structure of protein [34], [35]. On interaction of EC as well as ECG with BSA, decrease in ellipticity was observed in the CD spectrum of BSA, indicating partial unfolding of the helical structure. The α-helicity deferred from 58.1% in free BSA to 53.5% in BSA-EC complex and 58.8% in free BSA to 49.9% in BSA-ECG complex (Table 3). In the light of these results it can be reasoned that the α-helical content of BSA decreased on interaction with ECG to a greater extent than EC, with a slight increase in the β-sheet form. From the CD analysis, it can be inferred that the galloyl moiety induces stronger binding with the BSA resulting in higher unfolding at a lower D/P ratio compared to EC.

### FT-IR Spectra Study

Fourier transform infrared spectroscopy has long been used as a powerful method for investigating the secondary structures of proteins and their dynamics [36]–[39]. In the infrared region, the frequencies of bands due to amide-I and amide-II vibrations are sensitive to the secondary structure of proteins. The amide-I (C = O stretching) and amide-II (C–N stretching coupled with N–H bending) peak positions occur in the region 1650–1654 cm^−1^ and 1548–1560 cm^−1^ respectively [40]. Amide-I band is useful for the secondary structure studies as it is more sensitive to the change of protein secondary structure than amide-II [41]. The FT-IR spectra of native BSA (1×10^−4^ M) is shown in Fig. 7A, where the IR bands of amide-I and amide-II are distinct. The effect on secondary structure of BSA due to complex formation with catechins is summarized in Fig. 7B–7E. The amide-I peak position shifts from 1654 cm^−1^ in free BSA to 1655 cm^−1^ in case of BSA:EC complex (1∶1) and 1656 cm^−1^ for BSA:ECG complex (1∶1). Increasing the concentration of EC keeping BSA concentration same (1∶2) did not result in further peak shift. However, in infrared spectrum of BSA-ECG complex (1∶2) further peak shift of amide-I to 1657 cm^−1^ was observed. In the amide-II region also slight peak shift from 1544 cm^−1^ to 1543 cm^−1^ was observed for both (1∶1 and 1∶2) complexes. CD as well as FT-IR results unravel that ECG when bound to BSA brings about changes in secondary structure mainly in the α-helix which are more pronounced than those observed for EC-BSA complex.

### Characterization of Catechin-BSA Interaction by Microcalorimetry

In this work, we have used isothermal titration calorimetry in determining binding affinity, enthalpy, entropy and stoichiometry of binding of catechins with BSA. Representative isothermal titration calorimetric heat profiles for the binding of EC and ECG with BSA at pH 6.7 and 25°C are shown in Fig. 8A and 8B, respectively. Each peak in the binding isotherm represents a single injection of the catechin into the protein solution. The thermodynamic parameters associated with the binding of EC (500 µM) and ECG (500 µM) with BSA (50 µM) are summarized in Table 4. From Fig. 8A and 8B it is observed that the titration of BSA with EC and ECG yielded negative heat deflection. The integrated heat profile shown in the bottom panel of Fig. 8A and 8B for EC and ECG is corrected for all the dilution effects. The binding of EC and ECG to BSA is observed to be exothermic process with binding constants 9.68×10^4^ M^−1^ and 2.7×10^5^ M^−1^, respectively, as determined by the following equation:
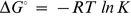
(4) where Δ*G*° is the free energy, *R* and *T* are the gas constant and temperature, respectively, *K* is the binding constant. The value of the stoichiometry of the binding is fixed to one based upon the goodness of the fit to ITC data and fluorescence observations vide supra. From Table 4, it is evident that the binding of EC and ECG to BSA is an exothermic process accompanied by positive values of Δ*S*° and a negative value of Δ*G*°. The binding process is always spontaneous as evidenced by the negative value of Δ*G*°. For typical hydrophobic interaction both Δ*S*° and Δ*H*° are positive, which are not observed in the present findings. The positive entropy observed accounts for hydrophobic interactions and negative enthalpy may play a role in electrostatic interactions which is also inferred from the decrease in quenching constant with increase in ionic strength. Furthermore, *T*Δ*S*° contributes significantly to Δ*G*° signifying that the binding of the catechins EC and ECG is entropy driven.

### Conclusions

This paper provides a comparative approach for studying the interaction of bovine serum albumin with dietary flavonoids EC and ECG using different biophysical techniques. It is evident from this work that the ECG which is the galloylated form of EC interacts strongly with BSA. The galloyl moiety has three phenolic and a carboxyl group which contribute substantially to the higher antioxidant activity and higher binding efficiency of ECG to BSA compared to EC. The binding forces are established to be hydrophobic and electrostatic in nature and stabilized with hydrogen bonding. ECG with antioxidant activity comparable to gallic acid, combined with high affinity for blood carriers can be used as a model drug. This report thus has a huge significance in the field of pharmacology and clinical medicine.
